# Functional and genetic characterization of clinical malignant hyperthermia crises: a multi-centre study

**DOI:** 10.1186/1750-1172-9-8

**Published:** 2014-01-16

**Authors:** Werner Klingler, Sebastian Heiderich, Thierry Girard, Elvira Gravino, James JA Heffron, Stephan Johannsen, Karin Jurkat-Rott, Henrik Rüffert, Frank Schuster, Marc Snoeck, Vincenzo Sorrentino, Vincenzo Tegazzin, Frank Lehmann-Horn

**Affiliations:** 1 Department of Neuroanesthesiology, Ulm University, Ludwig-Heilmeyer-Str. 2, Günzburg 89312, Germany; 2 Division of Neurophysiology, Ulm University, Albert-Einstein Allee 11, Ulm 89081, Germany; 3 Department of Anesthesiology and Intensive Care Medicine, Hannover Medical School, Carl-Neuberg-Strasse 1, Hannover 30625, Germany; 4 University of Basel, Basel, Switzerland; 5 University of Naples, Naples, Italy; 6 Biochemistry Department, University College Cork, Cork, Ireland; 7 Department of Anesthesia and Critical Care, University of Würzburg, Würzburg, Germany; 8 Rare Disease Center, University Hospital Ulm, Ulm 89081, Germany; 9 University of Leipzig, Helios Kliniken Leipziger Land Leipzig, Germany; 10 Department of Anesthesia, Canisius-Wilhelmina Hospital, University of Nijmegen, Nijmegen, The Netherlands; 11 Molecular Medicine Section, Department of Molecular and Developmental Medicine, University of Siena, via A. Moro 2, Siena 53100, Italy; 12 Department of Anesthesia, University of Padua, Padua, Italy

**Keywords:** Malignant hyperthermia, Succinylcholine, Suxamethonium, Volatile anesthetics, RyR1 mutations, In vitro contracture test

## Abstract

**Background:**

Malignant hyperthermia (MH) is a rare pharmacogenetic disorder which is characterized by life-threatening metabolic crises during general anesthesia. Classical triggering substances are volatile anesthetics and succinylcholine (SCh). The molecular basis of MH is excessive release of Ca^2+^ in skeletal muscle principally by a mutated ryanodine receptor type 1 (RyR1). To identify factors explaining the variable phenotypic presentation and complex pathomechanism, we analyzed proven MH events in terms of clinical course, muscle contracture, genetic factors and pharmocological triggers.

**Methods:**

In a multi-centre study including seven European MH units, patients with a history of a clinical MH episode confirmed by susceptible (MHS) or equivocal (MHE) in vitro contracture tests (IVCT) were investigated. A test result is considered to be MHE if the muscle specimens develop pathological contractures in response to only one of the two test substances, halothane or caffeine. Crises were evaluated using a clinical grading scale (CGS), results of IVCT and genetic screening. The effects of SCh and volatile anesthetics on Ca^2+^ release from sarcoplasmic reticulum (SR) were studied in vitro.

**Results:**

A total of 200 patients met the inclusion criteria. Two MH crises (1%) were triggered by SCh (1 MHS, 1 MHE), 18% by volatile anesthetics and 81% by a combination of both. Patients were 70% male and 50% were younger than 12 years old. Overall, CGS was in accord with IVCT results. Crises triggered by enflurane had a significantly higher CGS compared to halothane, isoflurane and sevoflurane. Of the 200 patients, 103 carried RyR1 variants, of which 14 were novel. CGS varied depending on the location of the mutation within the RyR1 gene. In contrast to volatile anesthetics, SCh did not evoke Ca^2+^ release from isolated rat SR vesicles.

**Conclusions:**

An MH event could depend on patient-related risk factors such as male gender, young age and causative RyR1 mutations as well as on the use of drugs lowering the threshold of myoplasmic Ca^2+^ release. SCh might act as an accelerant by promoting unspecific Ca^2+^ influx via the sarcolemma and indirect RyR1 activation. Most MH crises develop in response to the combined administration of SCh and volatile anesthetics.

## Background

Malignant hyperthermia (MH) is a rare autosomal dominant pharmacogenetic muscle disorder. The genetic incidence is thought to be between 1:3,000 and 1:8,500 [1]. Predisposed individuals are at risk of developing a severe drug-induced hyper-metabolic state resulting from altered Ca^2+^ turnover in the skeletal muscle. Volatile anesthetics and succinylcholine (SCh) are the classical triggering agents. The principal clinical symptoms are hypercapnia, acidosis, generalized muscle rigidity, cardiac arrhythmia and high temperature [1]. These clinical symptoms are used within a clinical grading scale (GCS) to predict the probability of whether a clinical event might be an MH crisis [2].

**Figure 1:**
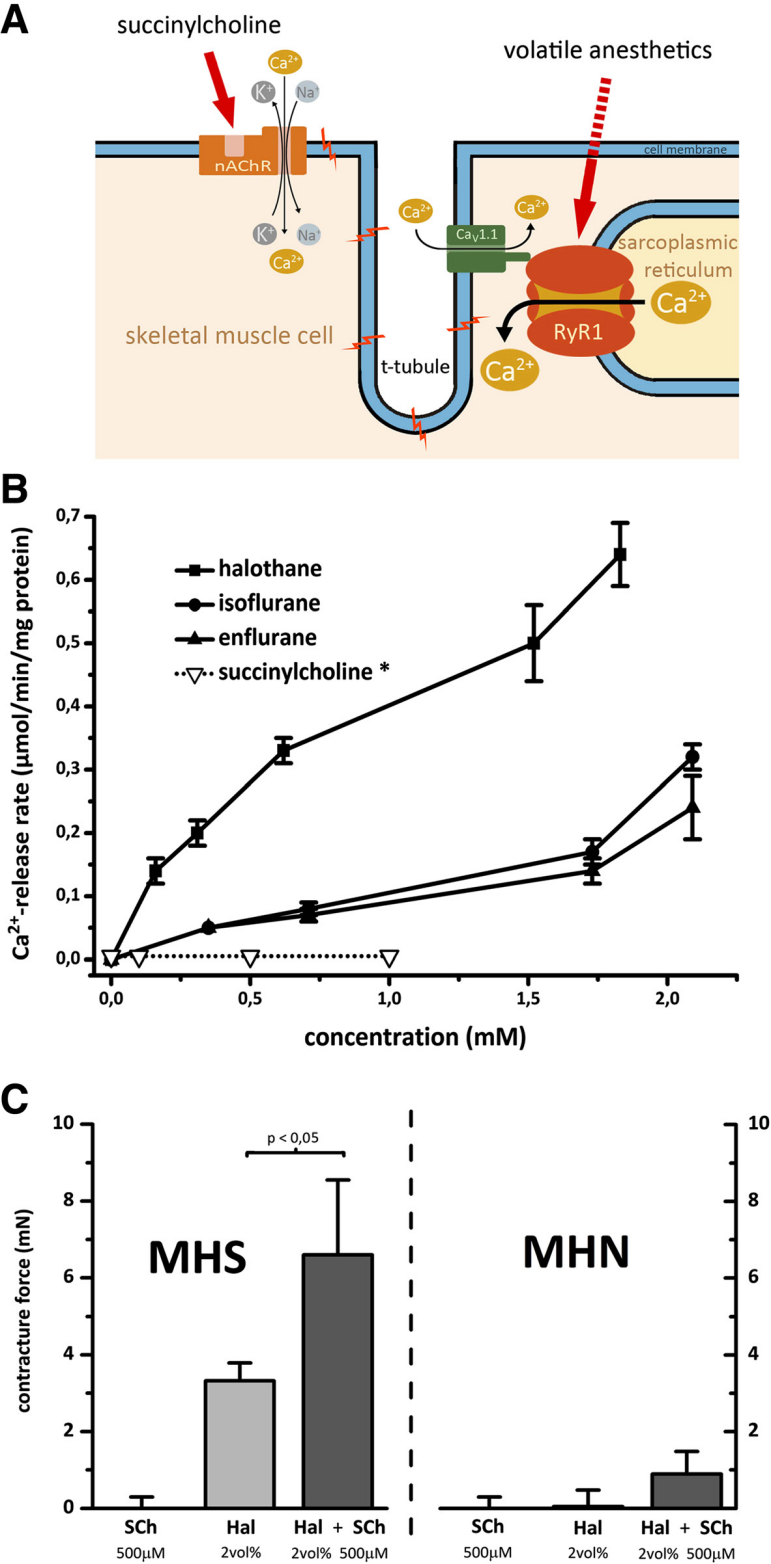
**Effects of MH triggers on Ca**^**2+ **^**release. A**: Uncontrolled myoplasmic Ca^2+^ release is the key to malignant hyperthermia. The most prominent cytosolic Ca^2+^ elevation results from the freeing of stored sarcoplasmic Ca^2+^ mediated by ryanodine receptor type 1 (RyR1). While volatile anesthetics stimulate Ca^2+^ release via RyR1, succinylcholine acts indirectly by activating the nicotinergic acetylcholine receptor (nAChR), a nonspecific cation channel, resulting in continuous local depolarisation. The depolarization can trigger propagated action potentials and will further activate the dihydropyridine receptors (DHPR, Ca_V_1.1) leading to the gating of both Ca^2+^ release from the SR via RyR1 and L-type Ca^2+^ current from the extracellular space. **B**: Heavy SR from rat muscle was maximally preloaded with Ca^2+^ before testing the potential Ca^2+^ releasing agonists halothane, isoflurane, enflurane and succinylcholine. The resulting Ca^2+^ release is via the RyR1 channel. Halothane, isoflurane and enflurane induced Ca^2+^ release from the SR vesicles but succinylcholine had no detectable effect. Results are expressed as mean ± standard error from six separate SR specimens. Of the three anesthetics tested, halothane showed the greatest potency and efficacy.* **C**: Succinylcholine (SCh) significantly increases halothane induced contractures in malignant hyperthermia susceptible individuals. However, SCh alone does not lead to the development of pathological contractures in MHN or MHS individuals*. *Part of the data from Figure 1B and C was published in Klingler et al. in 2005 [25].

In skeletal muscle, the primary mode of Ca^2+^ release is through direct protein-protein interaction between the voltage sensor of the t-tubular membrane, the dihydropyridine -sensitive L-type Ca^2+^-channel Ca_V_1.1 (DHPR) and the ryanodine receptor subtype 1 (RyR1), the Ca^2+^ release channel of the sarcoplasmic reticulum (SR) (Figure 1A). The RyR1 is identified as a key element in the pathophysiology of MH [3,4]. Currently more than 300 different variants of uncertain significance in the gene coding for RyR1 have been detected, however until now only 31 RyR1 mutations have been proven to be causative for MH according to the criteria of the European Malignant Hyperthermia Group (see http://www.emhg.org). In very rare cases, a defect in the α1-subunit of the DHPR has been reported [5], yet in up to 40% of the MHS families no mutations in either of the two genes could be identified [6,7]. The genetic penetrance is not fully understood because acute MH episodes are more common in males and children [8].

Muscle of individuals with a RyR1 mutation exhibits an increased sensitivity to volatile anesthetics: in vitro, MH muscle is more sensitive to halothane compared to other volatile anesthetics [9-12], however clinical studies have yielded inconsistent conclusions [13-15]. The MH diagnostic in vitro contracture test (IVCT) measures abnormally strong contractures as a surrogate marker for halothane or caffeine induced Ca^2+^ release from the SR [16]. MH susceptibility is a clearly autosomal dominant in vitro. The depolarizing muscle relaxant succinylcholine (SCh) pharmacologically activates the nicotinergic acetylcholine receptor (nAChR) which acts as a nonspecific cation channel resulting in a local long-lasting inward current and corresponding depolarization of the cell membrane. Since the nAChR is permeable to Ca^2+^, in addition to the depolarisation the entry of Ca^2+^ may indicate the underlying mechanism of action of SCh in the pathogenesis of MH. Acute crises triggered by SCh may be caused either by a direct influx of extracellular Ca^2+^ via the nAChR, by transient depolarization of the voltage-gated DHPR or by unspecific Ca^2+^ influx such as store-operated Ca^2+^ entry and Ca^2+^ entry coupled with excitation [17].

In vitro studies could not show that SCh alone induces pathological contractures in MH muscle strips although it did enhance the effect of halothane [18] and caffeine [19]. In vivo models are inconsistent with the in vitro findings: Harrison showed that MH pigs developed an increase in body temperature and muscle rigidity after administration of SCh alone [20] and in a recently developed micro-dialysis pig model, halothane and not SCh induced a hyper-metabolic reaction [21]. Using 284 suspected clinical MH cases, Dexter et al. demonstrated an increased risk of MH when SCh is used in combination with volatile anesthetics while SCh alone was only rarely seen as a trigger [22]. Additionally, pharmacological SCh formulations used to contain the preservative 4-chloro-m-cresol (4-CmC) which has been identified as a potent RyR1 agonist [23]. It was subsequently removed from SCh formulations in the 1980s [24].

The pathophysiology and variability of the clinical course of MH is not fully understood. A genetic approach is compromised due to numerous mutations of unclear causality, epigenetic factors and the involvement of at least two different Ca^2+^ conducting proteins. Functional testing (e.g. IVCT, Ca^2+^ release experiments) can only clarify partial aspects of the pathomechanism of MH. The goal of this study is thus to characterize a large cohort of proven clinical MH events by comparing CGS with functional effects on excised muscle bundles (IVCT) and genetic factors. We were also interested in the MH specific differences in the *in vitro* and *in vivo* characteristics of the classical MH triggers, i.e. SCh and volatile anesthetics, as these drugs act on different pharmacological pathways.

## Methods

### Multi-centre evaluation

Seven European MH test units participated in this multi-centre study: Basel, Switzerland; Nijmegen, Netherlands; Naples, Italy; Leipzig, Germany; Padua, Italy; Ulm, Germany; and Würzburg, Germany. Patients were included if they suffered from a clinical MH episode confirmed by a positive or an equivocal IVCT. These experimental investigations were part of the routine diagnostic algorithm used in MH susceptibility testing. Written informed consent was obtained from the patients or their legal guardians. Data collected included age, gender, year of crisis, clinical grading scale (CGS), information of the administration of MH trigger substances and results of the IVCT. All data sets were imported into a multi-centre database.

### Clinical grading scale

The MH episodes of the patients were analyzed by calculating the clinical grading scales (CGS) according to Larach et al. 1994 [2]. In brief, the CGS is a scoring system estimating the likelihood of an MH event using several indicators: masseter spasms or generalized muscle rigidity (process I: rigidity), maximum serum creatine kinase (CK) or maximum serum myoglobin levels (process II: muscle breakdown), maximum PaCO_2_ (process III: respiratory acidosis), maximum temperature (process IV: temperature increase), tachycardia or ventricular arrhythmia (process V: cardiac involvement), negative base excess (BE), arterial acidosis, and rapid reversal of MH signs after IV dantrolene (other indicators). The highest score of the processes noted are added together. The resulting raw scores are assigned as MH ranks which helps to predict the likelihood of an acute MH crisis: MH rank 1: “almost never”, MH rank 2: “unlikely”, MH rank 3: “somewhat less than likely”, MH rank 4: “somewhat greater than likely”, MH rank 5: “very likely” and MH rank 6: “almost certain”. Only datasets were included in which all necessary parameters for the calculation of CGS were measured and available.

### In vitro contracture test

Biopsies were taken from the vastus medialis or lateralis of the quadriceps muscle under regional anesthesia or trigger-free general anesthesia. The muscle specimens were used to perform IVCTs using the regularly revised protocol of the European Malignant Hyperthermia Group (EMHG) [16]. This test is used to determine the sensitivity of the skeletal muscle to caffeine and halothane which in turn allows assessment of the predisposition to MH. The viable specimens were kept at 37°C, pH 7.4 in Krebs-Ringer solution (mmol L^-1^: NaCl 118.1; KCl 3.4; CaCl_2_ 2.5; MgSO_4_ 0.8; KH_2_PO_4_ 1.2; NaHCO_3_ 25.0; glucose 11.1) and dissected into individual strips 15 mm to 25 mm length, 2 mm to 3 mm in width and 100 mg to 300 mg in weight. Muscle strips with twitch amplitudes greater than 10 mN to supra-maximal electrical stimulation (pulse frequency of 0.2 Hz; pulse width of 1 ms), or a contracture of ≥ 50 mN in the caffeine test in response to 32 mmol L^-1^ caffeine were considered viable. The force elicited was detected by a myo-electrical transducer (e.g. the FT03 from Grass Instruments, Quincy, MA). Pathological contractures were defined as having a threshold of 2 mN occurring at concentrations of 2 mmol L^-1^ caffeine or less or 2% (v/v) (0.44 mmol L^-1^) halothane or less. Individuals with pathological contractures related to both caffeine and halothane were considered to be MH susceptible (MHS); patients whose specimens developed pathological contractures to only one test drug were considered to be MH equivocal (MHEc for caffeine positive, MHEh for halothane positive), and if no pathological contractures occurred at 2 mmol L^-1^ caffeine and 2% (v/v) halothane the patient was classified as MH negative (MHN). Surplus tissue from muscle biopsy specimens of the Ulm MH unit was used in further analysis with the approval of the local ethics committee (Ethics Committee of Ulm University). The contracture forces following a pharmacological challenge with 500 μmol SCh, 2% (v/v) halothane, and the combination of both substances was studied. Halothane was purchased from Zeneca (Planckstadt, Germany), and preservative-free SCh was purchased from Curamed Pharma (Karlsruhe, Germany). Further details are provided in Klingler et al. 2005 [25].

### Genetic screening

Blood samples of the patients were genetically screened for RyR1 mutations of all 106 RYR1 exons and additionally for known mutations of CACNA1S. The CACNA1S gene encodes for the α1-subunit of the L-type Ca^2+^ channel Ca_V_1.1. Briefly described, blood cells were haemolysed and then DNA was extracted and amplified by polymerase chain reaction for further analysis. Details of the method are described in Zullo et al. 2009 [26]. Three different prediction algorithms were used to estimate a possible impact of novel amino acid substitutions on structure and function of RyR1: SIFT (http://sift.jcvi.org/), *Mutation taster* (http://www.mutationtaster.org/), and *Polyphen2* (http://genetics.bwh.harvard.edu/pph2/).

### Ca^2+^ Release in isolated SR

Heavy SR was prepared from hind limb muscles of rats as previously described [25,27]. Ca^2+^ release was measured by spectrophotometry using a HP 8452A diode-array spectrophotometer operating in dual wavelength mode at 710 nm and 790 nm at 37°C with constant magnetic cuvette stirring. Isolated SR was incubated with the Ca^2+^ chelometric dye antipyralazo III in a total volume of 2 ml in a ground glass-stoppered glass cuvette using a medium containing 19 mmol L^-1^ MOPS, 93 mmol L^1-^ KCl, 7.5 mmol L^-1^ sodium pyrophosphate, 1 mmol L^-1^ MgATP, 5 mmol L^-1^ creatine phosphate, 20 μg/ml CK and 250 mmol L^-1^ antipyralazo III at pH 7.0. Ca^2+^ flux was monitored continuously over time, and when the SR was maximally loaded with Ca^2+^ potential releasing agents were added at varying concentrations to establish whether Ca^2+^ release occurred. Succinylcholine, halothane, isoflurane and enflurane were added to the cuvette from concentrated stock solutions made up in pure ethanol using a gas-tight micro-syringe. Controls showed that ethanol had no effect at the concentrations used. SR protein concentration was measured as previously reported [25,27]. Anesthetic concentrations in the cuvette reaction medium were analyzed using electron capture gas chromatography. The three anesthetics were first purified by distillation [27].

### Statistical analysis

Results are presented as a mean with standard deviation. Discrete data are also provided as median and interquartile range (25% to 75%), black horizontal lines within the boxes show median values, whiskers indicate ranges and white squares represent mean values. Differences between the groups were assessed using the non-parametric Mann–Whitney U-test (also known as Wilcoxon rank-sum test or Mann–Whitney-Wilcoxon) and results were interpreted as significant if p < 0.05.

## Results

### Differing in vitro effects of volatile anaesthetics and succinylcholine

In a first set of experiments, we investigated different sub-cellular action sites within muscle fibres (Figure 1A). We analysed isolated SR-vesicles and found that volatile anesthetics stimulate SR mediated Ca^2+^ release: Isolated heavy SR of rat muscle strips revealed a significant increase in Ca^2+^ after administration of halothane, isoflurane and enflurane. In contrast to the effects observed with volatile anesthetics, SCh did not affect Ca^2+^ release from isolated SR vesicles at concentrations of up to 1 mmol L^-1^ (Figure 1B). Myographic recordings show that preservative-free SCh at concentrations of up to 1 mmol L^-1^ does not evoke contractures in isolated muscle bundles. There was however a significant contracture increase when SCh was combined with halothane or caffeine (Figure 1C).

### Multi-centre evaluation

Seven European MH test units participated in this multi-centre evaluation. The data set included 263 patients. In total 63 of them had to be excluded from the study: 60 of these were due to incomplete initial clinical documentation, three of them because of possibly interfering co-morbid factors: one being an intensive care patient with malaria and pneumonia, one being a polytrauma patient with hereditary sensorimotor neuropathy type 1 (Charcot-Marie-Tooth disease) and one being a King-Denborough patient with a non-anesthetic event. The remaining 200 cases were included – 165 of them MHS and 35 MHE. These crises happened during the time period from 1972 to 2010; patients were subsequently transferred to one of the investigation units of this multi-centre study for diagnosis. In five patients central cores were identified histologically. All of them carried RyR1 mutations of unknown causality (p.R4735E, p.I2453T, p.I4138T, p.D60Y, p.E342K). The histological examination yielded non classifiable core like lesions in another patient. She carried the RyR1 mutation p.R44C and suffered a severe clinical crisis (CGS = 78 points).

There was only one conclusive MHS patient whose MH crisis was triggered by SCh in the absence of volatile anesthetics. This 13 years old boy developed a masseter spasm and generalized muscle rigidity after induction with thiopental and intubation with SCh during ENT surgery; dantrolene was not given. He later showed a peak creatine kinase of 17,768 U/L. The calculated CGS was 15 points (rank 3: “somewhat less than likely”). The IVCT showed an abnormal reaction (MHS) and genetic analysis revealed a causative RyR1 mutation (p.R614C). Similarly one MHE patient was triggered by SCh alone: This 10 years old boy underwent emergency surgery because of testicular torsion. After application of SCh without pre-curarization clinical signs compatible with MH were masseter spasm and increased body temperature (40°C) (CGS = 25 points, rank 4 “somewhat greater than likely”). The IVCT was abnormal for caffeine (MHEc); no RyR1 mutation was detected.

**Figure 2:**
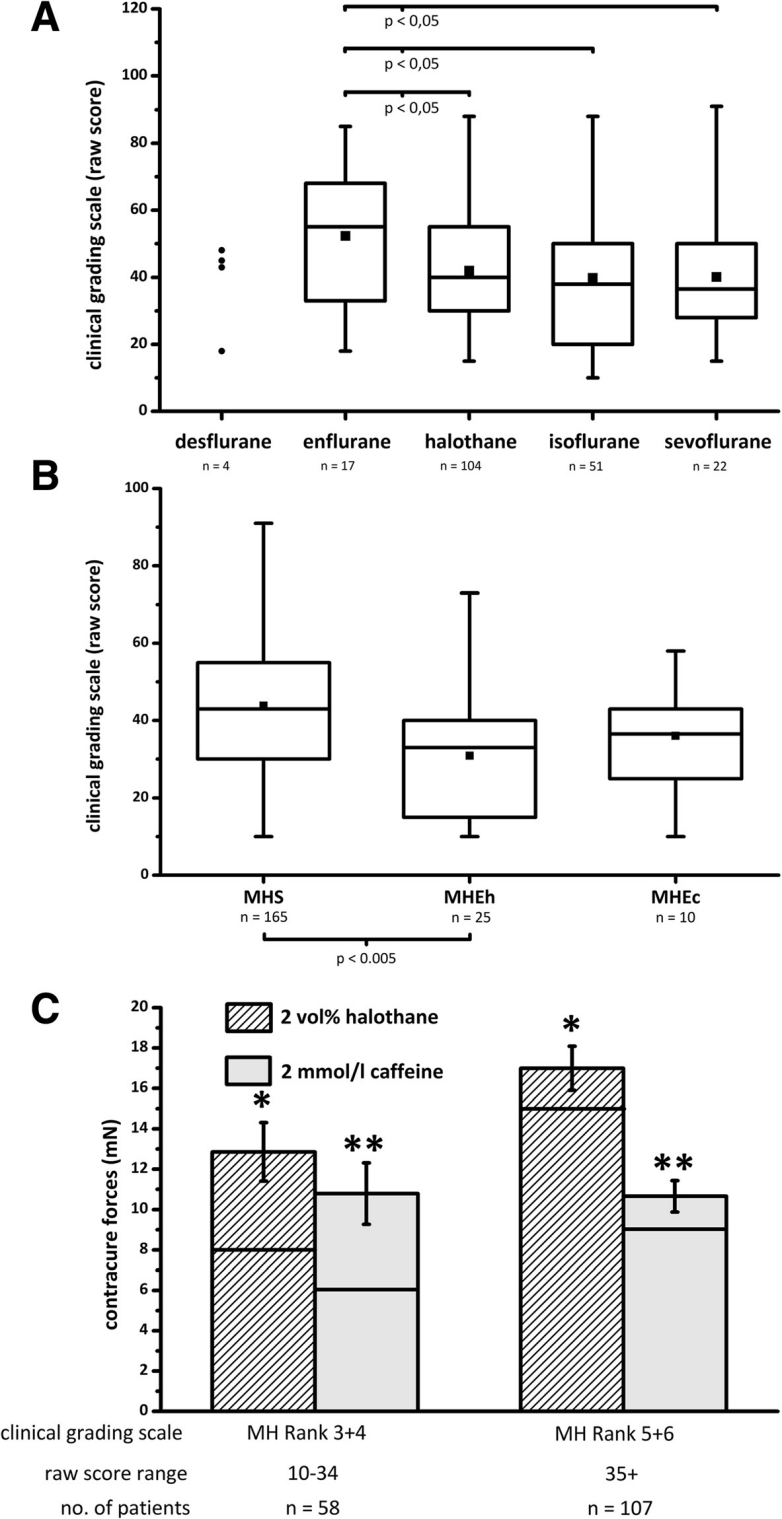
**Clinical effects of volatile anesthetics. A**: Box and whisker plots showing clinical grading scales (CGS) of 200 malignant hyperthermia susceptible (MHS, n = 165) or equivocal (MHE, n = 35) patients depending on the anesthetic agent used. Enflurane developed a significantly higher CGS compared to halothane, isoflurane and sevoflurane. **B**: CGS depending on the in vitro contracture test results: malignant hyperthermia susceptible (MHS), malignant hyperthermia equivocal halothane positive (MHEh) and caffeine positive (MHEc). A Mann–Whitney U-test was performed and yielded significant differences between MHS vs. MHEh, i.e. MHS vs. (MHEh + MHEc). **C**: Patients in this study with clinical crises that resulted in high MH Ranks (5 and 6) developed greater halothane and caffeine contractures than patients with lower MH Ranks (3 and 4). Asterisks (*, **) indicate significant differences. Columns represent mean ± standard error of the mean and black horizontal lines within the columns show median values.

**Figure 3:**
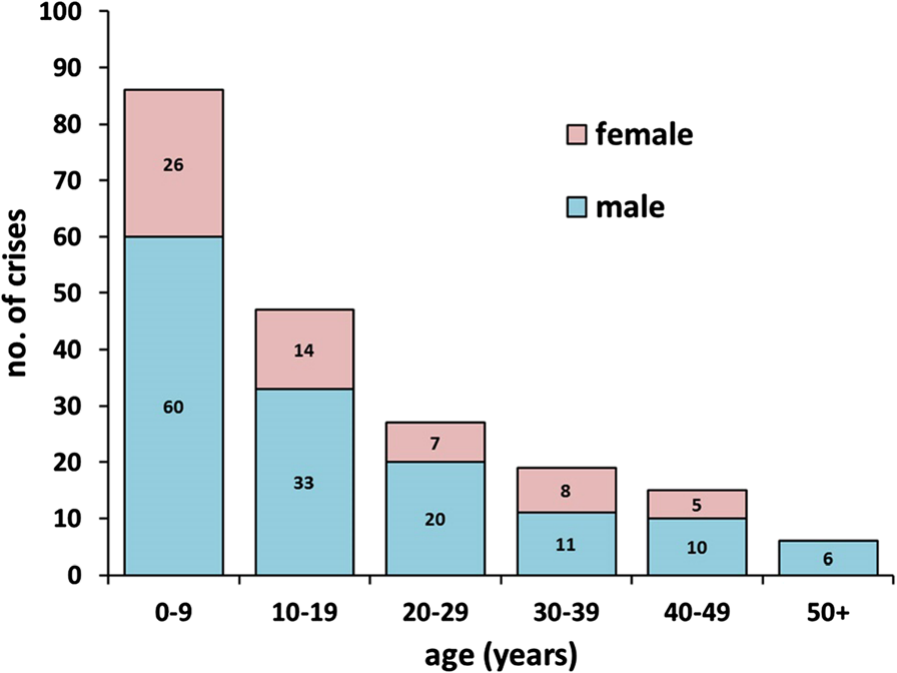
**Age and gender preponderance.** Age and gender of 200 MH patients at the time of the clinical MH-episode.

In the majority (MHS = 81%, MHE = 80%) both volatile anesthetics and SCh were administered. In the other cases (MHS = 18%, MHE = 17%) patients had received volatile anesthetics alone (Table 1). A Mann–Whitney U-test was performed which showed no significant difference in the raw score of CGS between patients who received volatile anesthetics alone and those who received volatile anesthetics plus SCh. The enflurane subgroup showed a significantly higher CGS compared to halothane, isoflurane and sevoflurane (Figure 2A).The age of the halothane group (10.5 ± 10.4) was significantly younger compared to the age of those receiving desflurane (40.5 ± 18.7), enflurane (19.7 ± 11.1), isoflurane (27.2 ± 15.6) and sevoflurane (20.5 ± 12.8). Patients classified as MHS showed a significantly higher CGS (43.8 ± 19.6) compared to those tested MHE (32.3 ± 14.5) (Figure 2B), even though the distribution of halothane and enflurane cases were similar in both subgroups (halothane 6.07 vs. enflurane 6.33). The IVCT and CGS results showed consistent results: MH ranks 5 and 6 developed significantly higher contractures and significantly lower thresholds compared to MH ranks 3 and 4 (Figure 2C). Half of the patients (50%) were younger than 12 years old at the time of crises and males (70%) were more often affected than females (30%) (Figure 3), however the CGS and the IVCT parameters did not differ significantly between males and females or adults and children.

**Table 1:** Multicenter evaluation of triggering potency

**Trigger**	**No. of patients**		**Clinical grading scale**	
			**(raw score)**	
	**MHS**	**MHE**	**MHS**	**MHE**
**Vol. anesthetics**	30	6	40.5 (28.5 - 61.0)	34.0 (30.8 - 41.0)
**SCh**	1	1	15	25
**Vol. anesthetics + SCh**	134	28	43.0 (30.0 - 55.0)	33.0 (15.0 - 40.0)
**Total**	**165**	**35**	**43.0 (30.0 - 55.8)**	**33.0 (19.0 - 40.0)**

The vast majority of the cases were triggered by the combination of volatile anesthetics and succinylcholine (SCh). Remarkable only one MHS case was triggered by SCh alone, along with one MHE case. The clinical grading scale according to Larach et al. 1994 classifies a raw score of more than 35 as very likely to be clinical MH. Data are shown as median and interquartile range (25% - 75%).

### Genetic evaluation

**Figure 4:**
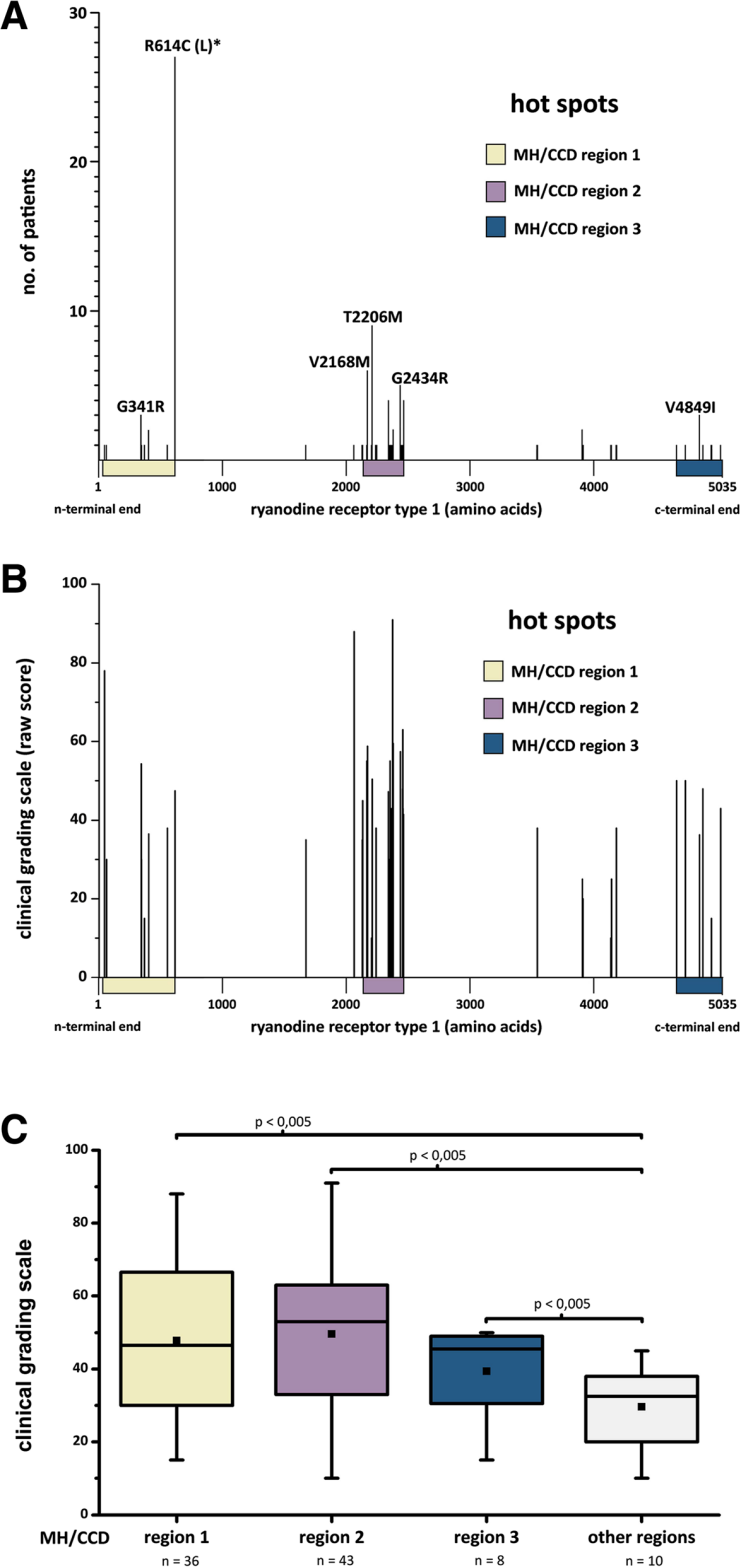
**Locations and effects of ryanodine receptor type 1 mutations. A**: Amino acid (AS) sequence of the ryanodine receptor type 1 (RyR1) from the n-terminal end to the c-terminal end. Most of the mutations found in this study are located in one of the three hot spots: MH/CCD region 1: AS 35 to 614; MH/CCD region 2: AS 2163 to 2458; MH/CCD region 3: AS 4664 to 5020. **B**: Clinical grading scale (mean) for each RyR1 mutation in regard of the location of the patients mutation within the gene. **C**: Box plot showing clinical grading scales (CGS) depending on the location of the ryanodine receptor type 1 mutation. Boxes delineate the inter-quartile range (25% to 75%), black horizontal lines within the boxes show median values, whiskers indicate ranges and white squares represent mean values. Mann–Whitney U-test reveals significantly higher CGS of MH/CCD region 1, 2 and 3 compared to other regions of the protein.

The overall RyR1 variant detection rate was 52%; 51 different RyR1 mutations were detected in 101 patients (Table 2). Four patients carried two RyR1 mutations (Table 3). Overall 14 new RyR1 variants are described in this study. Results of *SIFT*, *Mutation taster* and *Polyphen2* analysis is shown in Tables 2 and 3. Two patients carried RyR1 polymorphisms: exon 15, c.1655G > A, p.R552Q (new variant, personal communication with V. Sorrentino) and exon 38, c.6178G > T, p.G2060C [6] which occurs in 6% of the European population according to GeneCards. One MHS patient showed a nonsense mutation in exon 103 (c.14833C > T, p.R4945X, novel variant, K. Jurkat-Rott). Stop codon mutations like R4945X have been identified in several MH families but they never segregated with the MHS status in the given family. One patient showed a Ca_V_1.1 mutation (exon 4, c.520C > T, p.R174W); further statistical analysis was therefore not possible. Four patients did not give permission for genetic screening and therefore had to be excluded from genetic analyses. Most of the RyR1 mutations were found inside the “hot spots” (MH/CCD regions 1, 2 and 3) (Figure 4A). The halothane and caffeine contractures were both significantly higher if the mutation was found in one of these hot spots. Consistently, the thresholds of both test substances were significantly lower in hot spot mutations and these patients showed higher raw scores in the CGS (Figure 4B,C).

**Table 2:** Mutations of ryanodine receptor type 1

				**In vitro contracture test**									
				** Contracture**	**Threshold**								
**Exon**	**Nucleotide**	**Substitution**	**No. of patients in this study**	**2 vol% halothane [mN]**	**2 mmoll**^**-1 **^**caffeine [mN]**	**Halothane [vol%]**	**Caffeine [mmoll**^**-1**^**]**	**Clinical grading scale**	**Causative mutation?**	**PolyPhen2 predictions**	**Sift predictions**	**Mutation Taster predictions**	**Reference**
2	c.130C>T	p.R44C	1	12.0	10.8	0.5	1.0	78.0	No				Tammaro et al. 2003 [28]
**3**	**c.178G>T**	**p.D60Y**	**1**	**13.0**	**4.5**	**1.0**	**2.0**	**30.0**	**No**	**+**	**+**	**+**	**This study, V. Sorrentino**
11	c.1021G>A	p.G341R	3	14.3 ± 4.8	13.7 ± 3.1	0.8 ± 0.2	0.8 ± 0.5	54.3 ± 4.9	Yes				Quane et al. 1994 [29]
**11**	**c.1024G>A**	**p.E342K**	**1**	**37.8**	**23.8**	**0.5**	**0.5**	**30.0**	**No**	**+**	**+**	**+**	**This study, K. Jurkat-Rott**
11	c.1100G>A	p.R367Q	1	10.0	4.1	0.5	1.5	15.0	No				Galli et al. 2006 [30]
12	c.1201C>T	p.R401C	1	17.0	7.0	1.0	1.5	18.0	No				Davis et al. 2002 [31]
12	c.1202G>A	p.R401H	1	21.0	12.0	1.0	1.5	55.0	No				Rüffert et al. 2002 [32]
**15**	**c.1655G>A**	p.R552Q*	**1**	**36.0**	**8.0**	**0.5**	**1.5**	**38.0**	**No**	**+**	**-**	**+**	**This study, V. Sorrentino**
17	c.1840C>T	p.R614C	25	13.7 ± 8.9	10.5 ± 8.3	0.9 ± 0.5	1.3 ± 0.7	50.8 ± 22.3	Yes				Gillard et al. 1992 [33]
17	c.1841G>T	p.R614L	2	16.6 ± 2.6	8.3 ± 2.3	0.5 ± 0.0	1.0 ± 0.5	30.5 ± 2.5	Yes				Quane et al. 1997 [34]
**34**	**c.5011G>A**	**p.A1671T**	**1**	**8.0**	**24.8**	**2.0**	**0.5**	**35.0**	**No**	**+**	**+**	**-**	**This study, K. Jurkat-Rott**
38	c.6178G>T	p.G2060C*	1	16.4	8.0	0.5	1.0	88.0	No				Robinson et al. 2006 [6]
39	c.6377G>A	p.R2126Q	1	26.8	8.8	0.5	2.0	35.0	No				Kraeva et al. 2011 [7]
39	c.6387C>G	p.D2129E	1	10.0	11.0	2.0	1.0	45.0	No				Rüffert et al. 2001 [35]
39	c.6488G>C	p.R2163P	1	20.0	4.0	1.0	2.0	55.0	No				Robinson et al. 2006 [6]
39	c.6502G>A	p.V2168M	6	22.5 ± 7.1	12.3 ± 5.0	0.5 ± 0.0	1.1 ± 0.3	58.8 ± 20.5	Yes				Manning et al. 1998 [36]
40	c.6599C>T	p.A2200V	1	-	3.0	-	2.0	10.0	No				Sambuughin et al. 2005 [37]
40	c.6617C>T	p.T2206M	9	20.5 ± 10.7	10.4 ± 4.9	0.9 ± 0.4	1.0 ± 0.4	50.4 ± 16.2	Yes				Manning et al. 1998 [36]
**41**	**c.6710G>A**	**p.C2237Y**	**1**	**6.0**	**6.0**	**0.5**	**1.0**	**38.0**	**No**	**+**	**+**	**+**	**This study, M. Snoeck**
43	c.7007G>A	p.R2336H	4	12.8 ± 4.5	11.7 ± 6.1	0.8 ± 0.3	1.1 ± 0.2	47.3 ± 4.4	No				Levano et al. 2009 [38]
43	c.7025A>G	p.N2342S	1	3.0	0.0	2.0	4.0	30.0	No				Marchant et al. 2004 [39]
**44**	**c.7034G>C**	**p.S2345T**	**1**	**32.0**	**24.0**	**0.5**	**1.0**	**28.0**	**No**	**(+)**	**-**	**+**	**This study, V. Sorrentino**
44	c.7048G>A	p.A2350T	1	22.0	9.0	1.0	1.0	55.0	Yes				Sambuughin et al. 2001 [40]
**44**	**c.7076G>A**	**p.R2359Q**	**1**	**3.0**	**4.0**	**2.0**	**2.0**	**15.0**	**No**	**(+)**	**-**	**+**	**This study, H. Rüffert**
44	c.7085A>G	p.E2362G	1	16.0	8.0	0.5	1.0	43.0	No				Galli et al. 2006 [30]
44	c.7112A>G	p.E2371G	1	16.0	10.0	1.0	1.5	91.0	No				Zullo et al. 2009 [26]
44	c.7124G>C	p.G2375A	2	19.5 ± 0.5	20.5 ± 1.5	0.5 ± 0.0	0.8 ± 0.3	59.5 ± 11.5	Yes				Rüffert et al. 2002 [41]
45	c.7300G>A	p.G2434R	5	24.3 ± 14.4	12.2 ± 8.2	0.7 ± 0.2	1.1 ± 0.6	57.4 ± 19.9	Yes				Sambuugghin et al. 2001 [42]
46	c.7354C>T	p.R2452W	1	8.0	20.0	1.0	1.5	48.0	No				Chamley et al. 2000 [43]
46	c.7358T>C	p.I2453T	1	7.0	7.0	1.0	1.5	63.0	No				Rüffert et al. 2002 [41]
46	c.7360C>T	p.R2454C	1	9.2	6.0	0.5	1.0	28.0	Yes				Brandt et al. 1999 [44]
46	c.7361G>A	p.R2454H	3	15.3 ± 5.7	13.0 ± 6.5	0.8 ± 0.2	1.0 ± 0.4	48.0 ± 12.2	Yes				Barone et al. 1999 [45]
46	c.7372C>T	p.R2458C	2	7.3 ± 1.3	2.0 ± 1.0	1.0 ± 0.0	2.0 ± 0.0	41.5 ± 31.5	Yes				Manning et al. 1998 [46]
71	c.10616G˃A	p.R3539H	1	7.0	8.0	2.0	1.5	38.0	No				Dekomien et al. 2005 [47]
85	c.11708G>A	p.R3903Q	2	4.8 ± 0.2	2.5 ± 0.5	2.0 ± 0.0	2.0 ± 0.0	25.0 ± 5.0	No				Galli et al. 2006 [30]
**85**	**c.11723A>T**	**p.N3908I**	**1**	**8.0**	**4.8**	**1.0**	**1.5**	**20.0**	**No**	**+**	**+**	**+**	**This study, K. Jurkat-Rott**
**90**	**c.12398A>G**	**p.E4133G**	**1**	**2.0**	**2.5**	**2.0**	**2.0**	**10.0**	**No**	**+**	**+**	**+**	**This study, V. Sorrentino**
90	c.12413T>C	p.I4138T	1	11.0	15.0	1.0	1.0	25.0	No				Robinson et al. 2006 [6]
**90**	**c.12532G>A**	**p.G4178S**	**1**	**32.0**	**8.0**	**0.5**	**1.5**	**38.0**	**No**	**+**	**+**	**+**	**This study, V. Sorrentino**
95	c.13990T>C	p.C4664R	1	20.0	4.0	1.5	1.5	50.0	No				Zullo et al. 2009 [26]
**98**	**c.14204G>A**	**p.R4735Q**	**1**	**7.0**	**5.0**	**1.0**	**1.5**	**50.0**	**No**	**+**	**-**	**+**	**This study, H. Rüffert**
101	c.14545G>A	p.V4849I	3	3.8 ± 3.1	3.3 ± 0.8	1.5 ± 0.5	2.0 ± 0.0	36.3 ± 8.5	No				Jungbluth et al. 2002 [48]
101	c.14627A>G	p.K4876R	1	14.0	14.0	0.5	0.5	48.0	No				Monnier et al. 2005 [49]
**103**	**c.14833C>T**	**p.R4945X**	**1**	**9.9**	**23.3**	**0.5**	**0.5**	**15.0**	**No**	**na**	**na**	**+**	**This study, K. Jurkat-Rott**
**106**	**c.15059G>C**	**p.W5020S**	**1**	**1.0**	**2.0**	**-**	**2.0**	**43.0**	**No**	**+**	**+**	**+**	**This study, V. Sorrentino**

Overview of all ryanodine receptor type 1 (RyR1) mutations that have been detected in this study. If more than one patient carried the same mutation results of in vitro contracture tests (IVCT) and clinical grading scales are shown as mean ± standard deviation. Patients with double RyR1 mutations are listed separately. Novel variations (n = 13) are highlighted **(bold)**. Polymorphisms (n = 2) are marked with asterisks (*). **Polyphen2**: **+** = probably damaging, (+) = possibly damaging, **-** = benign, **na** = not applicable to truncations; **Sift: +** = deleterious, **-** = tolerated, **na** = not applicable to truncations; **Mutation taster: +** = disease-causing; **-** = polymorphism.

**Table 3:** Double mutations of the ryanodine receptor type 1

									**In vitro contracture test**				
									** Contracture**		** Threshold**		
**No. of patients in this study**	**Exon**	**Nucleotide**	**Substitution**	**Causative mutation?**	**PolyPhen2 predictions**	**Sift predictions**	**Mutation taster predictions**	**References**	**2 vol% halothane [mN]**	**2 mmoll**^**-1 **^**caffeine [mN]**	**halothane [vol%]**	**caffeine [mmoll**^**-1**^**]**	**CGS**
**1**	**11**	**c.1100G>T**	**p.R367L**	**No**	**+**	**-**	**+**	**This study, T. Girard**	20.0	4.5	1.0	1.5	48
	65	c.9649T>C	p.S3217P	No				Levano et al. 2009 [38]					
**1**	8	c.677T>A	p.M226K	No				Robinson et al. 2006 [6]	53.0	24.0	0.5	0.5	38
	28	c.4024A>G	p.S1342G	No				Levano et al. 2009 [39]					
**1**	44	c.7085A>G	p.E2362G	No				Galli et al. 2006 [30]	56.0	57.0	0.5	0.5	35
	93	c.13513G>C	p.D4505H	No				Groom et al. 2011 [50]					
**1**	29	c.4178A>G	p.K1393R	No				Vukcevic et al. 2010 [51]	15.0	12.0	0.5	1.5	35
	98	c.14210G>A	p.R4737Q	No				Monnier et al. 2005 [49]					

In this study four patients carried a double mutation of the ryanodine receptor type 1 (RyR1). These patients had marked outcomes in the in vitro contracture tests but clinical grading scales were avarage (mean: 39.00 points). Due to the small number of cases a statistical analysis was not performed. Novel mutations (n = 1) are highlighted (bold). CGS = clinical grading scale.

Patients with causative RyR1 mutations (as defined by EMHG) developed greater contractures, lower thresholds and higher raw scores in the CGS compared to patients with RyR1 mutations of unknown causality; however despite obvious caffeine contractures, no significant differences were detected between patients with mutations of unknown causality and patients without a RyR1 mutation (Table 4). In 8 of 35 MHE patients, an RyR1 mutation has been identified.

**Table 4:** Effect of causative ryanodine receptor type 1 mutation

**Classification of RyR1 mutation**	**Clinical grading scale**	** Contracture (mN)**		** Threshold**	
		**2 vol% halothane**	**2 mmol l**^**-1 **^**caffeine**	**halothane (vol%)**	**Caffeine (mmol l**^**-1**^**)**
Causative	51.10 ± 20.67* +	16.77 ± 9.84 + *	10.94 ± 7.24*	0.81 ± 0.44 + *	1.14 ± 0.63 **+** *
Unknown causality	38.08 ± 17.46*	11.69 ± 8.99*	8.73 ± 6.90*	1.10 ± 0.58*	1.50 ± 0.64*
None detected	37.55 ± 16.90**+**	11.43 ± 10.90**+**	7.52 ± 10.02*	1.30 ± 0.83**+**	2.35 ± 7.70**+**

Causative ryanodine receptor type 1 (RyR1) mutations yield greater contractures, lower thresholds and higher raw score in the clinical grading scale (CGS). Results of 189 patients are shown as mean ± standard deviation, Mann–Whitney U test was performed and significant differences (p < 0.05.) were marked with asterisk (*) and cross **(+)**. Despite caffeine contractures there were no significant differences between *unknown causality* vs. *none detected*. RyR1 polymorphisms (n = 2), double RyR1 mutations (n = 4) and Ca_V_1.1 mutations (n = 1) are not included in this table.

## Discussion

### Age and gender preponderance

The CGS was designed as an indicator for the likelihood that a given anesthetic crisis is MH. However, if the anesthetist recognized the crisis early and consequently started treatment, the crisis might result in a deceptively low CGS. There may be other factors (e.g. hormonal effects) that influence the risk of developing an acute MH episode. Our result resembles in part the findings of Islander et al. 2007 [8] and Green Larach et al. 2010 [52]: children (50%) and males (70%) dominate the case numbers, although results of IVCT and CGS did not differ between males and females.

### RyR1 mutations

At present there are more than 300 single nucleotide polymorphisms of the RyR1 known, while only 31 variants are functionally characterized as MH causative (http://www.emhg.org). The severity of IVCT varies between individuals with different RYR1 mutations [53]. In this study we confirm these findings and provide evidence that the RYR1 variants also vary in the severity of the clinical MH episodes: the clinical events were significantly more severe in patients suffering from mutations inside MH/CCD regions 1, 2 and 3. *SIFT*, *Mutation taster* and *Polyphen2* were used to characterize the relevance of novel RyR1 variants. All three prediction algorithms favour a possible effect on the protein function for the amino acid substitutions p.D60Y, p.E342K, p.C2237Y, p.N3908I, p.E4133G, p.G4178S and p.W5020S. Therefore a causative association to MH is likely. However, functional Ca^2+^ release experiments are needed to confirm gain of RyR1 function needed for MH susceptibility. Including the 14 novel RyR1 variants, 38 patients carried RyR1 mutations that have not yet been functionally analyzed. Those variants of *unknown causality* did develop less severe MH crises compared to functional analyzed *causative* mutations. Interestingly, mutations of *unknown causality* did not differ in the CGS compared to patients with wild-type RyR1. The RyR1 mutation p.R4945X is unlikely to cause MH because it results in a non-functional protein product rather than a gain-of-function as required for causative MH Mutations.

### Volatile anesthetics

In this study enflurane produced the highest CGS: the differences compared to halothane, isoflurane and sevoflurane were significant. However, this tendency was not found in other studies [14,15] and might be biased by differential handling of the crises such as the rapidness of dantrolene administration. Most crises were triggered by halothane. This might be influenced by the fact that halothane has been in use over the longest time span. On the other hand, patients in this study who received halothane were significantly younger compared to those who received other volatile anesthetics. At this point the patient’s age can be considered to be confounding variable; even though the CGS did not differ between age groups. As a result, the high number of halothane cases may not lead to any conclusion regarding its relative triggering potency. Still, in rat muscle halothane was significantly more likely to cause RyR1 mediated Ca^2+^ release than enflurane. In the literature, halothane is almost uniformly considered to be the most potent MH trigger [9-13]. Nonetheless, using the onset time of clinical symptoms Allen et al. did not find significant differences between halothane and desflurane when analyzing 365 unconfirmed crises from the American MH registry [14], and similarly Hopkins did not find significant differences between halothane and isoflurane in 75 cases confirmed by a positive IVCT [15]. Furthermore the relative triggering potencies of the other volatile anesthetics vary markedly in the above cited publications.

MH crises triggered by desflurane are described but seem to happen rarely: for example during the years 1990 to 2005, only two such cases were referred to the UK MH unit in Leeds [15]. In our study, we note four additional desflurane crises (CGS raw score = 38.5 ± 12.0), each confirmed by an MHS result in the IVCT.

### Succinylcholine

SCh activates the nAChR which depolarizes the muscle membrane by acting as an ion channel permeable to K^+^, Na^+^ and also Ca^2+^[54]. The depolarisation triggers propagated action potentials initially; these rapidly cease due to the refractoriness. The remaining nAChR-mediated depolarization spreads some distance electrically along the fibre axis depending on the fibre’s cable properties. In the t-tubules, it activates the DHPRs (Ca_V_1.1) which may lead to both entry of Ca^2+^ from the extracellular space and (through mechanical coupling) opening of the RyR1 along with Ca^2+^ release [55]. Whereas SCh action activates the excitation-contraction coupling pathway, volatile anesthetics cross the membrane and stimulate RyR1. In rat muscle volatile anesthetics were able to induce RyR1 mediated Ca^2+^ release, but not SCh [25]. Surprisingly we did not observe differences in the CGS of crises triggered by a SCh only versus SCh and volatile anesthetics. However the onset of MH crises was significantly faster when volatile anesthetics were combined with SCh [56]. The fact that we observed a SCh associated clinical crisis in the absence of volatile anesthetics does not prove MH triggering because undetected genetic variations or conditions explaining SCh hypersensitivity cannot be excluded.

Still, a recent study revealed that in more than 50% of the suspected MH crises in North America usage of SCh was recorded, while SCh was present in only 5% to 10% of all anesthetic records. Although this study was investigating unconfirmed crises only, the authors were able to demonstrate that the usage of SCh enhances the risk of an MH crisis developing when volatile anesthetics are given. [22].

## Conclusions

The consistent results of IVCT and CGS show that there must be patient-associated factors that determine the severity of an MH reaction. Conversely clinical penetration is variable as the same patient can undergo anesthesia with triggering agents and not develop clinical signs of MH.

In this study, a large group of patients (n = 38) have uncharacterized RyR1 mutations. Statistical analysis showed that these patients did develop less severe contractures and higher thresholds in the IVCT as well as lower raw score in the CGS. We conclude that this group of RyR1 mutations of *unknown causality* consists of both *causative* mutations still lacking proof of causality and non-causative RyR1 variants (polymorphisms). Also the genetic data show that the severity of MH varies depending on the location of the RyR1 mutation within the protein. The clinical observations of this multi-centre study indicate that the nAChR pathway might have the weakest potential in triggering an acute MH crisis. The data show that nearly all proven MH episodes were triggered by a combination of volatile anesthetics and SCh (81%) or volatile anesthetics only (18%). Notably the SCh only case in this study happened to a patient who showed all patient related risk factors: he was male, young (12 years old) and carried the *causative* RyR1 mutation p.R614C located within MH/CCD region 2. He developed a CGS of 15 points, which represents a less severe event. An anesthetist should be aware of possible MH reactions to SCh in clinical practice and moreover should know that the combination of volatile anesthetics and SCh in particular is dangerous in predisposed individuals.

## Competing interests

The authors declare that they have no competing interests.

## Authors’ contributions

WK designed the multi-centre study, supervised the IVCT in the Ulm MH unit, and he also worked on the manuscript. SH helped to design the multi-centre study, collected clinical data from the Ulm MH unit, did statistical calculations, drew the figures, and he also worked on the manuscript. TG collected clinical data, carried out genetic screening and supervised the IVCT experiments of the Basel MH unit; and he also worked on the manuscript. EG collected clinical data, carried out genetic screening and supervised the IVCT experiments for the Naples MH unit; she likewise worked on the manuscript. JH carried out Ca^2+^ release experiments on isolated SR in rat muscle and worked on the manuscript. SJ collected clinical data, supervised the IVCT experiments of the Würzburg MH unit and worked on the manuscript. KJR carried out genetic screening at the Ulm MH unit, did the polyphene analysis and worked on the manuscript. HR collected clinical data, carried out genetic screening and supervised the IVCT experiments for the Leipzig MH unit; he also worked on the manuscript. FS collected genetic data, supervised the IVCT experiments of the Würzburg MH unit and worked on the manuscript. MS collected clinical data, carried out genetic screening and supervised the IVCT experiments of the Nijmegen MH unit; he also worked on the manuscript. VS carried out genetic screening at the Padova MH unit and worked on the manuscript. VT collected clinical data and supervised the IVCT experiments of the Padova MH unit; he too worked on the manuscript. FLH collected clinical data from the Ulm MH unit, supervised the multi-centre study, managed the Ulm MH database and worked on the manuscript. All authors read and approved the final manuscript.
